# Case Report: Three cases of clinically suspected viral myocarditis with recovery of left ventricular dysfunction

**DOI:** 10.3389/fcvm.2024.1345449

**Published:** 2024-05-07

**Authors:** Jonathan Brown, Hytham Rashid, Siva T. Sarva, Suhas Tatapudi, Jeby Kalathoor, Aswin Srinivasan, Michael Daniel, Syed Raza

**Affiliations:** ^1^ Department of Internal Medicine, HCA Houston Healthcare Kingwood/University of Houston, Houston, TX, United States; ^2^ Department of Critical Care, HCA Houston Healthcare Kingwood/University of Houston, Houston, TX, United States; ^3^ Department of Cardiology, HCA Houston Healthcare Kingwood/University of Houston, Houston, TX, United States

**Keywords:** myocarditis, non-ischemic cardiomyopathy, coxsackievirus, heart failure, cardiogenic shock

## Abstract

Viral myocarditis is an important cause of non-ischemic cardiomyopathy. Multiple clinical manifestations have been reported, including acute heart failure, cardiogenic shock, and ventricular arrhythmias. We present three patients with clinically suspected viral myocarditis causing acute heart failure. Serum coxsackievirus B antibodies were positive in all three patients. Each case resulted in significant clinical improvement with hemodynamic support and acute recovery of left ventricular ejection fraction. Despite an initial critical presentation concerning for cardiogenic shock, we highlight three cases of clinically suspected coxsackie myocarditis with an excellent short-term prognosis.

## Introduction

Viral myocarditis is an important cause of acute cardiomyopathy resulting from inflammatory destruction of the myocardium (1). Enteroviruses, such as coxsackie B virus, are recognized as a common causal agent of acute myocarditis. Multiple clinical manifestations have been reported, including signs of acute heart failure, cardiogenic shock, and ventricular arrhythmias (2). The natural history of myocarditis is often unpredictable. Patients may experience an acute recovery of left ventricular systolic function, develop chronic and irreversible cardiomyopathy, or face fulminant disease resulting in acute cardiogenic shock and death. The diagnosis of clinically suspected myocarditis relies on a compatible clinical presentation with noninvasive diagnostic findings (3). Cardiac biomarkers including troponin I and troponin T may be elevated in cases of myocarditis, with increased levels associated with a worse prognosis (4). Electrocardiogram changes include sinus tachycardia, atrioventricular block, nonspecific ST-wave and T-wave abnormalities, and ST-segment elevation mimicking an acute myocardial infarction (5). Echocardiographic findings vary depending on the timing of the patient's presentation, often demonstrating new segmental or global wall motion abnormalities (3). Second-level investigations include cardiovascular magnetic resonance (CMR), coronary angiography to exclude ischemic cardiomyopathy, and endomyocardial biopsy (EMB). CMR serves as a highly specific, noninvasive diagnostic tool to characterize myocardial tissue, with the ability to detect inflammation, necrosis, fibrosis, and edema (1). Limitations of CMR include its inability to detect the degree of myocardial inflammation and cannot be used to exclude viral myocarditis (1, 3). Although EMB remains the gold standard for a definitive diagnosis, it is not routinely performed in patients with a clinical suspicion of myocarditis due to the lack of clinical value for guiding treatment or determining prognosis except in unique circumstances (6). Herein, we report a series of three cases with clinically suspected viral myocarditis.

## Case presentation

### Patient 1

**Figure 1:**
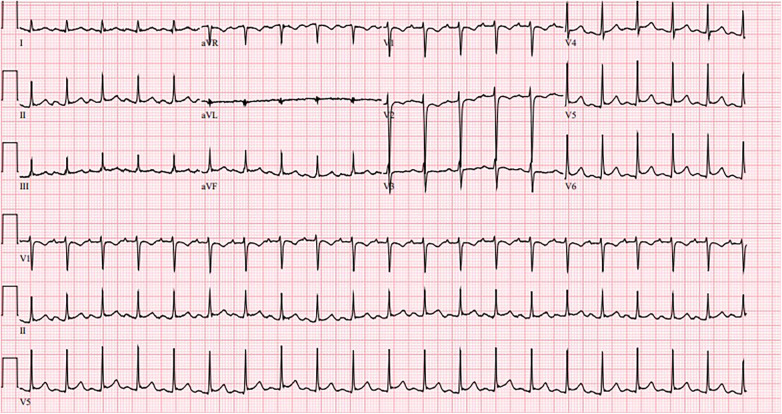
Sinus tachycardia without significant ST segment or T wave changes.

A 19-year-old male with no significant past medical history presented with five days of persistent nonbilious, non-bloody vomiting and worsening abdominal pain. Initial vital signs were significant for a temperature of 98.2°F, blood pressure of 80/47 mmHg, heart rate of 124/min, and oxygen saturation of 93% on room air. Electrocardiogram (EKG) showed sinus tachycardia without significant ST segment or T wave changes (Figure 1). Initial troponin was elevated at 2.15 ng/ml (*N*: < 0.012 ng/ml). Computed tomography (CT) of the abdomen without contrast was unremarkable. CT of the chest without contrast showed bilateral pulmonary edema. The patient was upgraded to the intensive care unit (ICU) due to suspected cardiogenic shock and started on vasopressors including norepinephrine and dobutamine. Echocardiogram showed a left ventricular ejection fraction (LVEF) of 20%–24% with severe global hypokinesis. Reverse transcription polymerase chain reaction for SARS-CoV-2 was negative. On day 3, the patient was weaned off vasopressors. Coronary angiography was deferred due to the absence of risk factors for coronary artery disease and the absence of ischemic changes on EKG. Serum coxsackievirus B (serotypes 1, 2, 5, and 6) IgG and IgM antibodies were positive. On day 6, the patient was initiated on a low dose of carvedilol and lisinopril. A repeat echocardiogram showed improvement of LVEF to 50%–54%. The patient had resolution of symptoms and he was stable for hospital discharge. Two weeks following hospitalization, the patient had achieved complete clinical recovery.

### Patient 2

**Figure 2:**
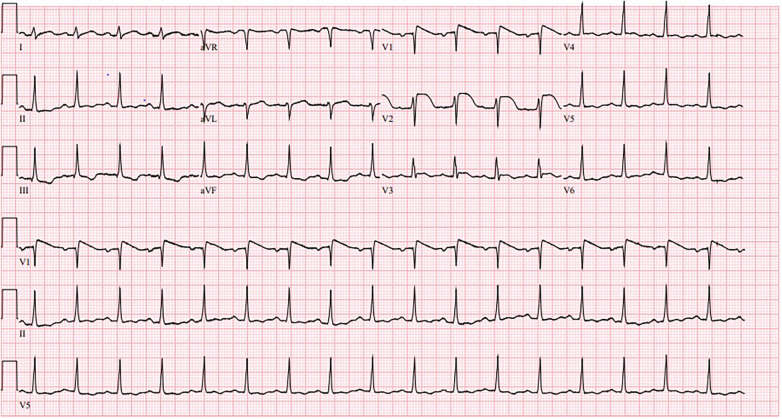
Normal sinus rhythm with ST segment elevations in leads V1 and V2.

A 20-year-old female with no significant past medical history presented with chest pain, dizziness, and epigastric pain. Initial vital signs were notable for a temperature of 98.2°F, blood pressure of 63/39 mmHg, heart rate of 106/min, and oxygen saturation of 100% on room air. Physical exam was benign except for abdominal tenderness in the epigastric region. The patient remained hypotensive and was admitted to the ICU for vasopressor support. EKG on admission showed acute ST segment elevations in leads V1 and V2 (Figure 2). Initial labs were notable for a creatinine of 4.6 mg/dl and an elevated troponin of 0.6 ng/ml (*N*: < 0.012 ng/ml). A transthoracic echocardiogram showed an LVEF of 25%–29% and diffuse hypokinesis with right ventricular dysfunction. The patient required dobutamine and norepinephrine for cardiogenic shock. Right and left heart catheterization with coronary angiography showed no significant coronary artery disease, normal pulmonary pressures, and normal cardiac output. On day 4, repeat EKG showed normal sinus rhythm with resolution of ST elevations, and the patient no longer required inotropic support. Serum coxsackievirus group B (serotypes 1–6) IgG and IgM antibodies was positive. Low dose sacubitril/valsartan and carvedilol was initiated on day 6 of hospitalization. Additional guideline directed medical therapy for heart failure was withheld due to low blood pressure measurements. She was stable for discharge on day 7 of hospitalization. Upon 1 month follow up, a repeat echocardiogram demonstrated a normal LVEF and no wall motion abnormalities.

### Patient 3

**Figure 3:**
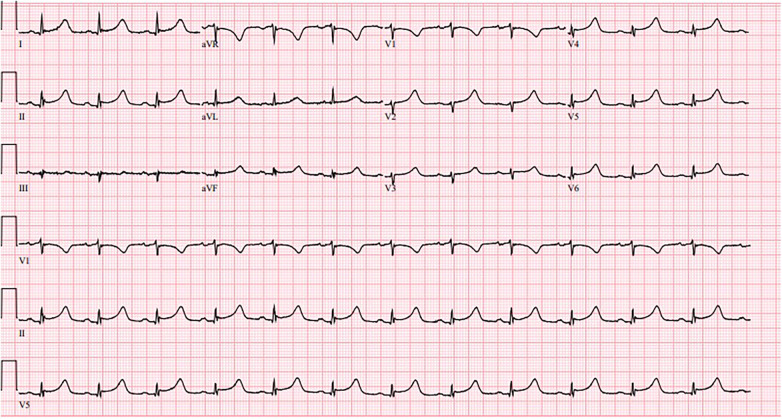
Normal sinus rhythm with nonspecific ST-segment changes.

A 78-year-old female with a history of hypertension, type 2 diabetes, and chronic hyponatremia presented with new onset dizziness and weakness. On admission, vital signs were notable for a temperature of 98.9°F, blood pressure of 70/40 mmHg, heart rate of 87 /min, and oxygen saturation of 99% on room air. Physical exam was notable for bilateral lower extremity pitting edema but otherwise unremarkable. EKG showed a normal sinus rhythm with nonspecific ST-segment changes (Figure 3). Initial troponin was 10.7 ng/ml (*N*: < 0.012 ng/ml). A transthoracic echocardiogram demonstrated a reduced LVEF of 40%–44% with hypokinesis of the anteroseptal and apical myocardium. A left heart catheterization showed twenty to thirty percent proximal and mid left anterior descending artery (LAD) stenosis which did not warrant intervention. Serum coxsackievirus group B (serotype 3) IgM and IgG antibody was positive. Hypotension improved with intravenous fluids. After nine days of hospitalization, the patient reported complete resolution of symptoms. She was continued on losartan and metoprolol with close outpatient follow up. Three months following admission, a repeat echocardiogram showed recovery of ventricular function with a LVEF of 60%–65%.

## Discussion

We highlight three unique cases of clinically suspected myocarditis with positive coxsackievirus B antibodies. Despite an initial presentation of cardiogenic shock, there was rapid clinical recovery within a week of hospitalization. The diagnosis of myocarditis relies on clinical presentations resembling acute coronary syndrome, nonspecific ST segment and T wave changes on ECG, followed by unexplained left ventricular dysfunction with either regional or global wall motion abnormalities on echocardiogram. Our first two cases consisted of healthy, young patients with no cardiovascular risk factors who prompted an investigation for new onset non-ischemic cardiomyopathy (NICM). In our third case, an elderly patient with multiple cardiovascular comorbidities, highly elevated troponin and nonspecific ischemic changes on ECG highlighted the importance of excluding coronary artery disease.

There were several limitations in our cases. First, endomyocardial biopsy was not performed during hospitalization due to the resolution of symptoms and improvement in LVEF within days of starting heart failure medical therapy. EMB is generally reserved for patients with new-onset fulminant myocarditis refractory to pharmacologic therapy or unexplained heart failure associated with a dilated left ventricle, ventricular arrhythmias, and atrioventricular block (6). In addition, CMR was not available at our hospital, and could have served as a valuable noninvasive tool to support the diagnosis of clinically suspected myocarditis. We recognize the limitation of serum coxsackie antibodies in the diagnosis of coxsackie myocarditis and that a definite diagnosis requires viral PCR on cardiac biopsy.

Myocarditis causes an inflammatory cardiomyopathy leading to acute heart failure with risk of hemodynamic instability, often requiring temporary inotropic and vasopressor support (7). Depending on the degree of left ventricular dysfunction, patients may experience rapid resolution, chronic dilated cardiomyopathy, or end stage heart failure with risk of sudden cardiac death (3). Myocarditis can result from multiple infectious pathogens including viruses, bacteria, fungi, and protozoans (2). Parvovirus B19, human herpesvirus 6 (HHV 6), and enteroviruses, including coxsackie B virus, are common viral etiologies of acute and chronic myocarditis (2). The pathogenesis involves introduction of the virus through the respiratory or gastrointestinal tract followed by entry into the myocardium (1). Direct infection of cardiomyocytes enables viral replication and activates immune responses, resulting in myocyte necrosis and cellular degradation (8). In addition, molecular mimicry between cardiac and viral antigens triggers an autoimmune reaction in which virus specific T cells target the myocardium. Over time, a chronic inflammatory response causes myocyte fibrosis and cardiac remodeling, resulting in dilated cardiomyopathy (2). Progressive ventricular dysfunction is associated with persistent viral infection as demonstrated from an EMB based analysis of clinically suspected viral myocarditis (9).

Echocardiography is essential in the initial work-up to exclude other causes of NICM such as amyloidosis or valvular heart disease. Echocardiographic features are nonspecific and encompass a wide spectrum of findings. They often demonstrate a reduced LVEF with global hypokinesis, as seen in two of our patients. Right ventricular dysfunction, shown to be a prognostic factor for mortality and the need for transplantation, was observed in one of our cases (2). Other potential features include increased septal thickening, regional hypokinesis, diastolic dysfunction, or pericardial effusion (10). Such findings on echocardiography cannot reliably distinguish myocarditis from acute coronary syndrome. Therefore, cardiac catheterization is warranted when coronary artery disease is suspected.

Treatment recommendations for viral myocarditis are based on expert consensus centered on guideline-directed medical therapy for heart failure. No clinically approved antiviral therapies are available for coxsackie B-related myocarditis due to the absence of randomized clinical trials in the field. However, anti-viral agents such as interferon-β (IFN-β) have gained recent attention due to cardiomyocyte protection and decreased inflammatory cell infiltration, as demonstrated in an experimental animal model (11). In addition, a phase-II study from Schultheiss et al. showed effective viral clearance, reduction in viral load, and improvement in NYHA functional class among patients receiving IFN-β in addition to standard heart failure therapy (12). The role of immunomodulatory therapy remains largely exploratory. Given the role of the coxsackievirus-adenovirus receptor for myocardial viral entry, neutralization with soluble virus receptor traps can inhibit development of chronic myocarditis and preserve cardiac function, as demonstrated in an animal model (13). Further randomized clinical trials are required to determine the effect of antiviral and immunomodulatory therapy on multiple clinical endpoints.

## Conclusion

We reported three cases of clinically suspected myocarditis requiring hemodynamic support. Serum coxsackie antibodies were positive in all patients. Patients hospitalized for left ventricular dysfunction secondary to acute myocarditis often have significant recovery within weeks. Clinical management for viral myocarditis centers on optimizing heart failure medical therapy. Given the possibility of progressive systolic dysfunction with viral persistence, close monitoring with long term follow up is required.

## Data Availability

The original contributions presented in the study are included in the article/**Supplementary Material**, further inquiries can be directed to the corresponding author.

## Supplementary material

Supplementary Video 1 Echocardiogram of patient 1, short-axis view showing reduced LVEF with severe global hypokinesis.

Supplementary Video 2 Echocardiogram of patient 2, short-axis view showing reduced LVEF with diffuse hypokinesis.

Supplementary Video 3 Echocardiogram of patient 3, long-axis view showing reduced LVEF with anteroseptal hypokinesis.
