# Supplementary material for: Case Report: Three cases of clinically suspected viral myocarditis with recovery of left ventricular dysfunction
Source: Front Cardiovasc Med. 2024 May 7;11:1345449. doi: 10.3389/fcvm.2024.1345449 (PMC11106496; doi:10.3389/fcvm.2024.1345449)
Supplement: Supplementary file 1 [file Table1.docx]

|  | Age | Presenting symptoms | Initial echocardiographic findings | Treatment on discharge | Length of Stay |
| --- | --- | --- | --- | --- | --- |
|  |  |  |  |  |  |
| Case 1 | 19 | nonbilious, non-bloody vomiting and worsening abdominal pain | left ventricular ejection fraction (LVEF) of 20-24% with severe global hypokinesis | carvedilol and lisinopril | 6 days |
| Case 2 | 20 | chest pain, dizziness, epigastric pain | LVEF of 25-29% and diffuse hypokinesis with right ventricular dysfunction | sacubitril/valsartan | 6 days |
| Case 3 | 78 | dizziness and weakness | LVEF of 40 to 44% with hypokinesis of the anteroseptal and apical myocardium | losartan and metoprolol | 9 days |
